# Stethoscopic Diagnosis of Pregnancy

**Published:** 1830

**Authors:** 


25. *Stethoscopic diagnosis of pregnancy.* The Boston Med. and Surg. Jour. March 23d inst. contains an interesting paper by Dr. Fisher, on auscultation in the diagnosis of pregnancy. He has had opportunities of testing the efficacy of this methed in 16 cases.

In applying the stethoscope over the womb of these individuals, I have heard in each two peculiar sounds; the one quick and rapid, resembling in some respects the ticking of a watch,—the other slow and prolonged, resembling the sound of a bellows. The former corresponded in every respect to the one which Kergaradec supposed to proceed from the action of the fœtal heart, and termed by him "the pulsation of the heart of the fœtus." The latter answers exactly to that which this author has termed a "simple blowing pulsation," or "placental sound," and which he supposes to arise from the placenta during the circulaton of blood through it.

In every one of the sixteen individuals that I have examined, I have heard distinctly and could count the pulsations of the heart of the fœtus. In about two-thirds of the cases, these pulsations were loudest and most distinct on the left of the linea alba, and in the left side of the womb. In a minority of the patients, the pulsations were most distinct in the opposite side of the womb and median line of the abdomen; and, in two or three instances, they were most audible immediately under the linea alba, and at the distance of two or three inches below the umbilicus. In which side soever of the womb or of the median line the child was, by the sense of touch, found to be situated, in that the pulsations of the fœtal heart were, in every instance, most audibly and extensively heard. In most instances, I experienced no difficulty in detecting these pulsations; —when the woman was very fleshy, however, or when the uterus contained an unusual quantity of liquor amnii, I was obliged, in order to recognise them perfectly, to press the instrument with considerable force upon the abdomen, and to command perfect silence in the room. With these precautions, I have never failed in hearing the heart's action whenever I have examined for it, and in being able to number its pulsations. The noise produced by the friction of the clothes, by the peristaltic motions of the bowels, or by some sudden muscular contraction of the patient, would indeed render them obscure for a moment, but it was only for a moment. The action of the fœtal heart resembles, in all respects save in frequency, that of the adult heart, and cannot be mistaken even by the unpractised ear. In the language of my notes written at the bedside, "the action of the fœtal heart is characterized by two sounds or pulsations; the pulsations are loud and clear, and those of the ventricles can be plainly distinguished from those of the auricles, almost as much so as in the adult heart. There is the gradual and deep sound of the contraction of the ventricle, which is immediately, and without interval, followed by the clear and valve-like sound of the contraction of the auricle;—a momentary rest of the organ now follows,—then occurs again the gradual and deep sound of the ventricle, followed by the clearer and sharper one of the auricle. There is no evident impulse communicated to the instrument by these pulsations, nor do I discover that the motions made by the mother in turning from side to side cause any variation in the pulsations of the fœtal heart. They continue to be regular and uniform during these movements, unless some convulsive motions of the fœtus happened at the same time, in which case they increase in rapidity."

The space over which these pulsations could be heard, their number, and the point of elevation in which they were strongest and most distinct, varied in different individuals and at different months of pregnancy. Generally speaking, they were audible over a space of seven or eight inches long, and five or six broad. In some, I have heard them from the groin to the naval, and even beyond it: and from the superior spinous process of the ilium of one side, to within two or three inches of that of the opposite side. In others, they were limited to an area of four or five inches. Their number varied from 118 to 155 in a minute, in different females and at different periods of gestation. In the earlier periods of pregnancy, they were more frequent than in the later periods. They are generally uniform and regular in their succession and number, but, when the child moves suddenly, or when any convulsive motions take place in its limbs, then the action of the heart becomes so rapid that it is difficult, and sometimes impossible, to count its pulsations. The spot where the pulsations were most audible, particularly during the last month of pregnancy, and under which the heart was most probably situated, was generally about three inches from the naval, on a line extending from it to the middle of the groin. This spot, however, varied in its situation in the different months, and when the patient turned from side to side.

Besides the stethoscopic phenomena now mentioned, I have heard, in most, but not in all of the cases stated, the "simple blowing pulsation" described by our French author, and which he denominates the "placental sound." The region in which, and the space over which, it could be heard, varies in different individuals and at different periods of gestation. I generally heard it more audibly in the upper and anterior portion of the womb, and almost always in a part of the organ opposite to that in which the fœtal pulsations were heard. If these were observed on the left side of the linea alba, then the placental sound was usually to be discovered on the right side of this line, and vice versa. In one instance, the sound proceeded from the lower part of the womb, near the public region; and, in a female yet to be confined, the sound can be heard on each side of the fundus of the womb, but cannot be distinguished in the central and most projecting part of it. In this last case, the placenta is probably attached to the upper and back part of the uterus, having the child immediately in front of it. The place of the placental sound was, in some cases, limited to three or four inches in extent; in others, and particularly in those whose abdomen was much distended by the waters of the ovum, the space over which it was audible was many inches in diameter.

The character of this sound is peculiar. It assumes, during the course of pregnancy, as Lænnec has observed, all the characters of the bellows-sound. About the commencement of the fifth month, which is the earliest period that I have had an opportunity of hearing it, the sound was characterized by a sort of rushing or rasplike noise, not unlike that produced by the action of a small file upon a thin soft board. Later in pregnancy, and during the eighth and ninth months, at which period I have heard and examined it most frequently, it is duller, and resembles very closely the sound produced by the blowing of a pair of bellows. This comparison, however, does not convey to the mind a pefect idea of the sound: it has more of a swelling tone or character, if I may so term it, than that produced by a pair of bellows: it resembles the noise of a broad dense flame, which is produced by the wind from a large pair of bellows, more than it does the simple issuing of the air from the bellows itself; or perhaps it still more perfectly resembles the noise of water as it is forced from the hose of a fire engine. This sound is always isochronous with the pulse of the mother, and varies in number and character with it. It increases and dies away in unison with the dilatation and contraction of the artery at the wrist, and its intensity was always the greatest at the moment that the impulse of the artery was greatest.

The placental sounds were always heard in the same part of the womb in the same individual, and were uniformly distinguished by the same peculiarities until the commencement of labour. But during the labour pains a new phenomenon takes place in regard to them. At the moment the pains occurred and the womb began to contract, I observed that the placental sounds became less sonorous and gradually died away as the pains increased, and finally, when these were most acute and the contraction of the uterus was greatest, they for a moment completely disappeared. When the pains are light and the contraction of the womb but partial, the placental sounds become less diffused and audible, but do not cease entirely.—

They diminish in intensity in proportion as the degree of pain and uterine contraction increases. As the pains cease and the uterus relapses again into its previous quiescent and dilated state, the placental sounds assume their accustomed character, and during the intervals of pain they vary in no respect from what they were before labor commenced. These phenomena I have heard repeatedly, and during many successive contractions of the womb. I have heard them in six different cases, and I want no other proof to convince me that the "simple blowing pulsation," or "placental sound" now described, proceeds from the placenta. But there are further proofs of this. The moment the child is born and the cord ceases to pulsate, the placental sound is no longer heard; and, in two cases, I have immediately, on the birth of the child, and before the cord was divided, passed my hand into the womb, and ascertained that the placenta was attached to that part of the organ from which the sound proceeded.

It is said that the placental sounds can be heard as early as between the third and fourth month of gestation, and immediately after the uterus has risen above the pelvis. The earliest period at which I have had an opportunity of examining for them, was the last of the fourth, or the commencement of the fifth month. They were audible at this period. They were occasionally intermittent, and could not during every examination be detected, until the last few weeks of pregnancy. During the ninth month I never failed hearing them distinctly whenever I searched for them. The friction of the clothes against the instrument, the noise made in the room, and the rolling of the intestines, would now and then overpower the sounds of the placental pulsations—but only for a short time. The moment these accidental causes were removed, the sounds would become as distinct and audible as ever.

In making the "external" examinations now mentioned, the female was made to lay upon her back, and to be lightly covered with clothes. The position of the patient, however, was often changed. Sometimes I made my examinations whilst the female was laying on her side,—on her face,—and whilst standing erect,—without, however, observing any very material variation in the situation or character of the stethoscopic phenomena.

I have never been able to hear the fœtal or placental pulsations by applying the stethoscope over the loins or sacrum, or over the back part of the abdominal walls. To ascertain the state of the womb by manual pressure, I usually commenced my "searching operations" at the symphysis pubes, and, from this point, I cautiously carried my fingers over every part of the abdomen. In using the stethoscope, I usually applied it, in the first instance, over the navel, so that this organ should be included within the open end of the instrument. From the navel, I moved it in every direction over the abdominal cavity. Proceeding in this manner, it was easy to discover in what region of the womb the pulsations were located, and the exact point where they were strongest and most powerful. Gen erally speaking, all these examinations may be made with as much facility as those of the chest can be, and with as little exposure of the patient, and without necessarily offending the delicacy even of the young and sensitive. Occasionally, however, in order to convince myself that I was not mistaken in the character of the sounds which I heard, I have been obliged to expose a part of the abdomen sufficiently extensive for the application of the instrument. But, in most cases, this was unnecessary. The phenomena were easily distinguishable through a simple covering of clothes, as a sheet, for instance. The covering, however, should be of linen or cotton, and, to prevent any noise which might arise from friction, I have found it useful to moisten the part of the covering over which the instrument was to be applied, or to dip the end of the instrument in water previous to its application. Either of these precautions will generally render any exposure of the abdominal surface entirely unnecessary.

The following inferences may justly be drawn, I think, from the preceding observations:—

1st. By means of the stethoscope it can generally, and perhaps always, be easily ascertained if the woman be pregnant with a living child; and whether she be pregnant with one or more living fœtuses.

2nd. By means of the same instrument the situation of the placenta can be determined, whether in the fundus, side, or over the mouth of the womb;—and—

3d. The situation of the child in the uterus may be distinguished, and the character of the presentation at birth may, at least in some cases, be foretold by means of external manual examinations.

